# Comparison of estimates of left ventricular ejection fraction obtained from gated blood pool imaging, different software packages and cameras

**DOI:** 10.5830/CVJA-2013-082

**Published:** 2014-04

**Authors:** Rachelle Steyn, John Boniaszczuk, Theodore Geldenhuys

**Affiliations:** Department of Nuclear Medicine, Groote Schuur Hospital, University of Cape Town, South Africa; Department of Nuclear Medicine, Groote Schuur Hospital, University of Cape Town, South Africa; Department of Nuclear Medicine, Groote Schuur Hospital, University of Cape Town, South Africa

**Keywords:** gated blood pool studies, cameras, software packages, results

## Abstract

**Objective:**

To determine how two software packages, supplied by Siemens and Hermes, for processing gated blood pool (GBP) studies should be used in our department and whether the use of different cameras for the acquisition of raw data influences the results.

**Methods:**

The study had two components. For the first component, 200 studies were acquired on a General Electric (GE) camera and processed three times by three operators using the Siemens and Hermes software packages. For the second part, 200 studies were acquired on two different cameras (GE and Siemens). The matched pairs of raw data were processed by one operator using the Siemens and Hermes software packages.

**Results:**

The Siemens method consistently gave estimates that were 4.3% higher than the Hermes method (*p* < 0.001). The differences were not associated with any particular level of left ventricular ejection fraction (LVEF). There was no difference in the estimates of LVEF obtained by the three operators (*p* = 0.1794). The reproducibility of estimates was good. In 95% of patients, using the Siemens method, the SD of the three estimates of LVEF by operator 1 was ≤ 1.7, operator 2 was ≤ 2.1 and operator 3 was ≤ 1.3. The corresponding values for the Hermes method were ≤ 2.5, ≤ 2.0 and ≤ 2.1. There was no difference in the results of matched pairs of data acquired on different cameras (*p* = 0.4933)

**Conclusion:**

Software packages for processing GBP studies are not interchangeable. The report should include the name and version of the software package used. Wherever possible, the same package should be used for serial studies. If this is not possible, the report should include the limits of agreement of the different packages. Data acquisition on different cameras did not influence the results.

## Abstract

Serial measurement of LVEF using gated blood pool (GBP) imaging is an established technique for monitoring LVEF in patients undergoing chemotherapy with cardiotoxic medication and in patients after heart transplants.^1^1,^2^ The nuclear medicine department at Groote Schuur Hospital performs up to a thousand GBP studies annually. The majority of these studies are for patients receiving cardiotoxic chemotherapy and have a significant impact on patient management.

In our hospital, the radiation oncologists consider not starting cardiotoxic chemotherapy if the LVEF is below 50% and terminating chemotherapy if there is a 10% decrease. In patients who have had heart transplants, the cardiologists start patients on glucocorticosteroids if a patient’s LVEF decreases by10%. It is therefore imperative that serial studies on an individual patient are comparable.

Two software systems are used in our nuclear medicine department. The Siemens system (Siemens Medical Solutions, Chicago, USA) was introduced in February 2006 and the Hermes system (Hermes Medical Solutions, Stockholm Sweden) in September 2007. After the introduction of the Hermes system, we found large differences between the LVEFs calculated by the two systems. This was confirmed by a pilot study and is consistent with the literature that different software programs for processing equilibrium gated radionuclide studies cannot be used interchangeably.^3^-^7^

The department also uses two different cameras, a General Electric (GE) Starcam 400 AC single-head and a Siemens Signature Series e.cam dual-head camera to acquire the raw data. These are then transferred to the Siemens and Hermes processing systems.

This study was done to determine how the software packages used for processing GBP studies should be integrated into our department and if the use of different cameras for acquisition influences results. The study had two components. The first examined the values and reproducibility of estimates of LVEF from two software packages using data acquired on the GE gamma camera and processed independently by three operators. The second component examined the values and reproducibility of estimates of LVEF calculated with the same software packages using matched pairs of raw data acquired on both gamma cameras (GE and Siemens) processed by one operator.

## Methods

Since October 2007 the raw data of all GBP studies done in the department have been stored in a Hermes electronic archive in the original format. These studies, acquired on the GE camera, were therefore available for reprocessing. All the patients were referred to our department as part of their diagnostic work-up.

The majority of studies were done for patients receiving cardiotoxic chemotherapy. A minority (< 5%) of the studies were done for patients who had heart transplants.

For the first component of the investigation, 200 studies acquired on the GE camera were selected using random-number tables to identify folder numbers of patient studies archived between 1 October 2007 and 15 July 2009. There were 1 952 studies performed on 1 473 patients during this period.

**Fig. 1.**
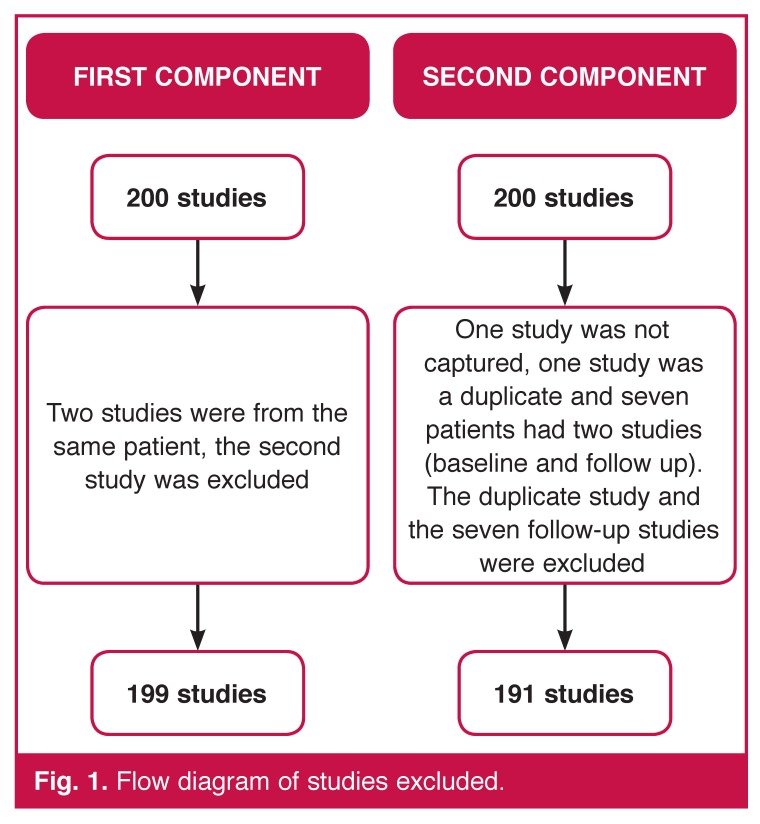
Flow diagram of studies excluded.

For the second component, 200 patients were studied. Two sets of data were acquired for each patient, the second immediately after the first. One of the sets was acquired on the GE and the other on the Siemens camera, the order depended on the availability of the cameras. This produced 200 matched pairs of data (Fig. 1).

Ethics approval for the study was obtained from the Research Ethics Committee, Health Sciences Faculty, University of Cape Town.

## Imaging protocol

An in-vivo method for labelling the red blood cells was used. One red blood cell labelling vial (Nuclear Technology Products, Pelindaba, SA) containing 20 mg sodium pyrophosphate and 4 mg tin dichloride was reconstituted with 5 ml NaCl and 3.5 ml was injected intravenously, followed 20 minutes later by an injection of 800–900 MBq of Tc-99m sodium pertechnetate eluted from a NovaTec P generator manufactured by NTP Radioisotopes (Pty) Ltd of Pelindaba. The dose administered was in accordance with the Society of Nuclear Medicine guidelines.^8^

For all patients, anterior, left lateral and left anterior oblique images were recorded in a 64 × 64 matrix with the patient supine. For the left anterior oblique image, the angle of the detector head relative to the patient was adjusted to give the best septal delineation. The ECG–RR interval was divided into 24 frames, the beat acceptance window set at 30% and the energy window at 15%.

A low-energy general purpose (LEGP) collimator manufactured by GE and zoom of 1.5 were used with the GE Starcam 400 AC single-head camera and acquisition was stopped when 8 000 kilocounts had been acquired. The GE camera was interfaced to an Alfanuclear acquisition system (IM512P Data and Image Processor version 2.0). A LEHR collimator manufactured by Siemens and a zoom of 2 were used on the Siemens Signature Series e.cam dual-head camera and acquisition was stopped when 8 000 kilocounts had been acquired. The Siemens camera was interfaced to a Siemens acquisition system (Version A4OA, Siemens Medical Solutions, Chicago USA).

## Processing

The studies were processed using the two methods available; one provided by Siemens (Gated Blood Pool Activity version 7.0.7.2, Siemens Medical Solutions, Chicago, USA) and one by Hermes (Functional Gated Analysis, FUGA version V4.7, Hermes Medical Solutions, Stockholm, Sweden ).

Semi-automated programs were used because the automated programs of both vendors placed the background region of interest (ROI) in the bottom left-hand corner of the field of view. This results in a background ROI that is not periventricular. It overlies the spleen, aorta or other soft tissue structures.

The default settings of the Siemens method was: a zoom of 2 was used; a Butterworth filter with a cut-off of 0.40 of the Nyquist frequency and order 5 was applied; the background ROI was placed on the end-systolic frame; X and Y shifts were 2 and the offset 4 pixels; height and width were 50%.

The default settings of the Hermes method was: no zoom was used; a Butterworth filter with a cut-off of 5 as defined by Hermes and order 70 was applied; the background ROI was placed on the end-diastolic frame.

In the first component of the study, the data acquired on the GE camera were processed three times by three independent operators. These were the senior author (operator 1), and two experienced radiographers (operators 2 and 3). The operators adjusted the position, shape and size of the background ROI.

While processing, the operators recorded the number of beats rejected, whether the labelling of the red blood cells was good, satisfactory or poor (this was done using visual analysis), whether the quality of the tracking of the left ventricle was good, satisfactory or poor (this was done using visual analysis), where the program placed the background ROI, where the operator placed the background ROI, the size of the background ROI, as well as the mean counts within it.

In the second component of the study, the data acquired on both the GE and Siemens cameras were processed three times by a single operator (operator 1) using the same software methods, default settings and intervention as for the first component.

## Statistical analysis

The results were entered into an EpiData version 3.1 database.^9^ Data were then exported for analysis into Microsoft Office 2007 Excel and STATA version 11.^10^

The Shapiro-Wilk test showed that the data were not normally distributed. Attempts at transformation were unsuccessful. (Tukeys ladder of transformations was used.) Parametric statistics were still applied however as it is deemed acceptable to apply parametric statistics if the number of subjects exceeds 30.

Means, standard deviations (SDs) and ranges (maximum and minimum) of estimates of LVEF were calculated. The Bland-Altman method of comparison analysis was used to assess the estimates of LVEFs as well as the impact of acquisition on different cameras. Analysis of variance was used to establish the statistical significance. The reproducibility of LVEFs was assessed using the SD of the three estimates of LVEF calculated by each operator for each method.

## Results

## Values and reproducibility of estimates of LVEFs

The left ventricle was not tracked in four studies when using the Siemens method. In all four studies the entire heart or the vascular structures above it were tracked. All three operators were in agreement in three of these studies. In one study only operator 3 was unable to track the left ventricle. The corresponding mean estimates of LVEF for these studies using the Hermes method were 36, 67, 66 and 74%.

With the Hermes method, the left ventricle was not tracked in one study. In this study the entire heart was tracked. Operators 1 and 2 were in agreement in this study; operator 3 however, was able to track the left ventricle. The corresponding mean estimate of LVEF for this study using the Siemens method was 63%.

These five studies were from different patients. The exclusion of the five studies left 194 studies for analysis.

**Fig. 2.**
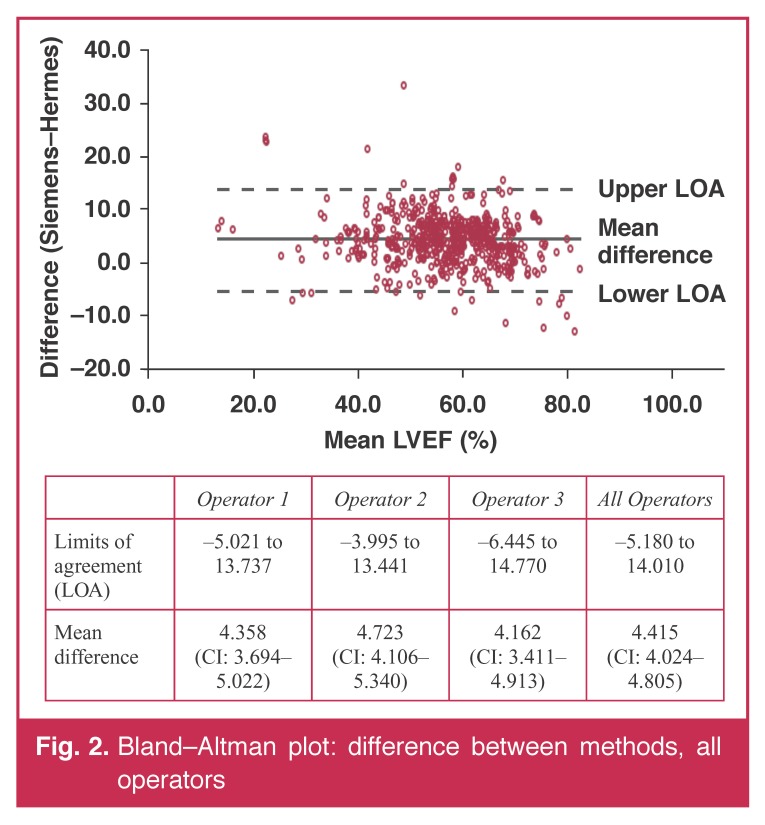
Bland–Altman plot: difference between methods, all operators

Table 1 summarises the values for the estimates of the LVEFs. There were no differences between the results obtained by the three operators but the Siemens method gave estimates that were 4.3% higher than those given by the Hermes method. The differences between the two methods were not related to the values obtained for the LVEFs, and the limits of agreement between the two methods were almost identical for all three operators (Fig. 2).

**Table 1:** Values of estimates of LVEFs; all operators

	*Siemens*			*Hermes*		
	*Mean (%)*	*SD*	*Range (%)*	*Mean (%)*	*SD*	*Range (%)*
Operator 1	59.1	10.1	19.3–82.0	54.8	11.0	11.0–88
Operator 2	59.5	10.1	18–82.3	54.7	11.0	10.0–82.3
Operator 3	58.8	10.3	16.7–82	54.6	11.4	10.0–85
All operators	59	10.2	16.7–82.3	54.7	11.1	10.0–88

There was a difference between methods (F 650, 54; df 1, 97; *p* < 0.0001) but no difference between operators (F 1, 72; df 2, 97; *p* = 0.18) and no interaction between operator and method (F 0, 90; df 2, 97; *p* = 0.41).

Of the five highest and five lowest estimates of LVEF obtained with each method by each operator, four of the highest five LVEFs and four of the lowest five LVEFs were from the same studies for each method by all three operators. Out of the five highest, three of the four were the same for both methods. Of the five lowest however, only one was the same for both methods.

Table 2 summarises the reproducibility of the estimates of the LVEFs. There were 53 patients in whom the SD of the three estimates of the LVEFs was above the 95th percentile for both methods for one or more operators. In most of these patients, two of the three estimates obtained by any one of the operators for a method were similar. The difference between these two similar estimates (minimum difference) was 0% in 14 patients, 1% in 26 patients, 2% in eight patients and 3% in five patients. The difference between the highest and lowest estimates (maximum difference) for all three operators for both methods was 3% in eight patients, 4% in 17 patients, 5% in 19 patients, 6% in six patients, 8% in two patients and 9% in one patient. The maximum difference for all three operators was 6% or less for the Siemens method and 9% or less for the Hermes method. The difference between the minimum and maximum estimates was not associated with any particular level of LVEF.

**Table 2:** Percentiles of the sds of the three estimates of LVEF for the Siemens and Hermes methods

	*Siemens method*			*Hermes method*		
	*Operator 1*	*Operator 2*	*Operator 3*	*Operator 1*	*Operator 2*	*Operator 3*
5th percentile	0.0	0.0	0.0	0.0	0.0	0.0
25th percentile	0.6	0.6	0.0	0.6	0.0	0.0
50th percentile	0.6	0.6	0.6	0.6	0.6	0.6
75th percentile	1.2	1.2	0.6	1.5	1.0	1.1
95th percentile	1.7	2.1	1.3	2.5	2.0	2.1

## Values and reproducibility of estimates of LVEFs acquired on two cameras

Both studies of one patient could not be processed because the left ventricle could not be tracked by either method. There were a further seven studies from five patients in which the data acquired on one of the cameras could not be processed by one of the methods. Of these studies, four were acquired on the GE camera and three on the Siemens camera.

For the studies acquired on the GE camera, the Siemens method tracked the heart and the left atrium in two studies (corresponding mean estimates of LVEF obtained by the Hermes method were 60 and 58%). The Hermes method tracked the heart and aorta in two studies (corresponding mean estimates of LVEF obtained by the Siemens method were 60 and 59%).

For the studies on the Siemens camera, the Siemens method tracked the left atrium and the aorta in two studies (corresponding mean estimates of LVEF obtained by the Hermes method were 63 and 61%, respectively), and the Hermes method tracked the entire heart in one study (corresponding mean estimates of LVEF obtained by the Siemens method was 60%). This left 185 patients for analysis.

**Fig. 3.**
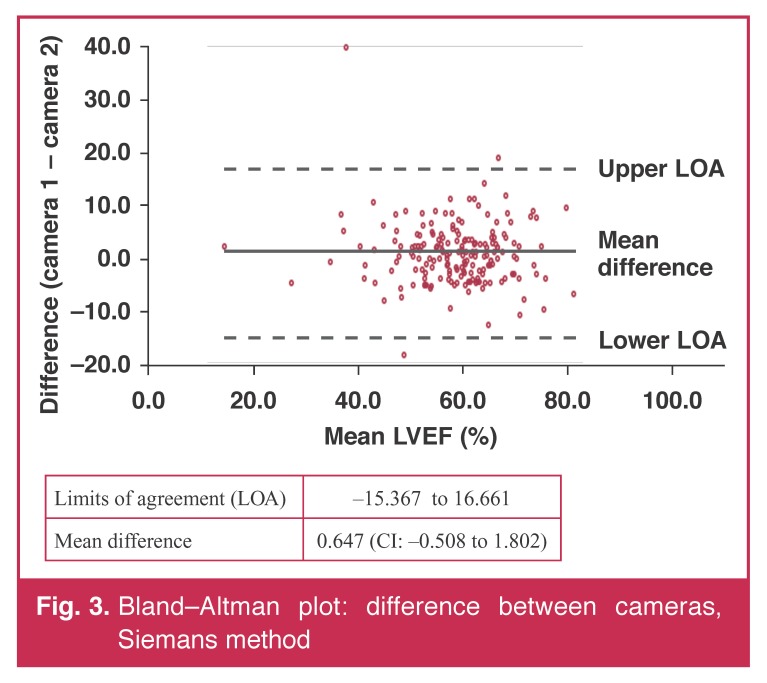
Bland–Altman plot: difference between cameras, Siemans method

**Fig. 4.**
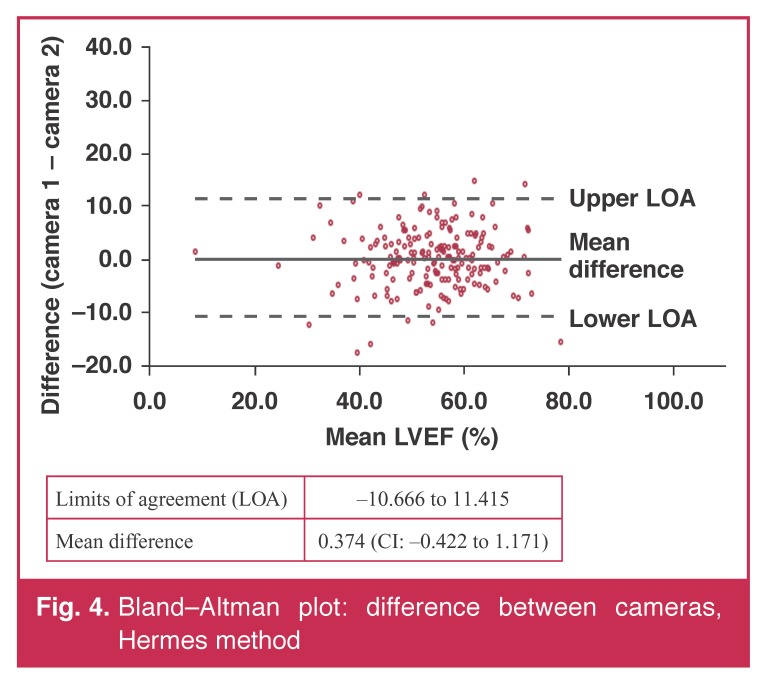
Bland–Altman plot: difference between cameras, Hermes method

Tables 3 and 4 summarise the values of the estimates of LVEF acquired on both cameras. There was no difference in the estimates. Bland–Altman plots (Figs 3 and 4) showed no bias in their distribution.

**Table 3:** Estimates of LVEFs acquired on different cameras processed by the Siemens method

*GE camera*			*Siemens camera*		
*Mean (%)*	*SD*	*Range (%)*	*Mean (%)*	*SD*	*Range (%)*
58.7	10.4	4.0–84.3	57.9%	10.3	13.3–84.7

There was no difference between acquisitions on different cameras (GE and Siemens) processed by the Siemens method (F 0, 47; df 1, 37; *p* = 0.49).

**Table 4:** Estimates of lvef s acquired on different cameras processed by the Hermes method

*GE camera*			*Siemens camera*		
54.3	10.2	9.3–79	53.9	10.1	7–86.3

There was no difference between acquisitions on different cameras (GE and Siemens) processed by the Hermes method (F 0, 0.8; df 1, 368; *p* = 0.77).

Table 5 summarises the reproducibility of the estimates of LVEF from data acquired on the two cameras. There were 40 patients in which the SDs of the three estimates of the LVEFs were above the 95th percentile for both methods on both cameras. In most of these patients, two of the three estimates obtained on one camera for a method were similar. The difference between the two similar estimates (minimum difference) was 0% in 14 patients, 1% in 10 patients, 2% in 10 patients, 3% in one patient, 4% in two patients, 5% in two patients, and 6% in one patient.

**Table 5:** Estimates of lvef s acquired on different cameras processed by the Hermes method

	*GE camera*		*Siemens camera*	
	*Siemens method*	*Hermes method*	*Siemens method*	*Hermes method*
5th percentile	0.0	0.0	0.0	0.0
25th percentile	0.6	0.6	0.6	0.6
50th percentile	0.6	1.0	0.6	1.0
75th percentile	1.2	1.7	1.2	1.5
95th percentile	2.3	3.0	3.1	2.9

The difference between the highest and lowest estimates (maximum difference) for both cameras for both methods was 1% in one patient, 4% in three patients, 5% in three patients, 6% in 14 patients, 7% in eight patients, 8% in four patients, 9% in three patients, 10% in one patient, 13% in one patient, 22% in one patient, and 33% in one patient. The differences of 22 and 33% were found in patients who were imaged on the Siemens camera and processed by the Siemens method. In both of these patients, it was documented by the operator that the tracking of the left ventricle was poor.

## Discussion

There is consensus in the literature that different software programs for processing GBP studies cannot be used interchangeably.^3^-^7^ This was also found in our study, which showed the Siemens software gave higher estimates of LVEF than the Hermes software. Hiscock *et al*,^3^ who compared 18 workstation algorithm combinations including Siemens and Hermes, also found that the Siemens software gave estimates of LVEFs that were higher than estimates obtained from the Hermes software. The mean of 64 estimates of LVEF calculated with the Siemens software was 64.6% and with the Hermes software 61.3 and 61.7%.

Differences in estimates of LVEF obtained from different software methods on the same patient could be attributed to variations in the algorithms used for edge detection in determining the ROI around the left ventricle and background subtraction. There was no documentation on the Siemens package available to us in the online user manual. There was limited documentation on the mathematical algorithms used in the Hermes software for determination of the left ventricular ROI and background subtraction. The Hermes software package used a variation on a second differential method for edge detection in determination of the left ventricular ROI. Background subtraction was performed in the background ROI outside the left ventricular ROI at the end of diastole, as well as on the non-ventricular counts within the end-diastolic region at end-systole. This could have resulted in an over-subtraction of background and account for a slightly lower LVEF.

In the studies performed by Skrypniuk^4^ and Fair *et al.*,^5^ it is suggested that there is a need for software suppliers to supply more information on their software packages and to give guidance on data quality requirements as well as on any limits of operation. Fair *et al.*^5^ suggested that adequate testing of software packages against phantoms (if possible), and clinical testing on a reasonable number of patients should be done by software manufacturers.

Because different software packages use different algorithms and give different values for LVEF, all reports of LVEF calculated from GBP studies should contain the name and version of the software package used to calculate the result. Wherever possible the same software should be used to process serial studies. When the same software is not used to process serial studies, ideally, all the raw data should be retrieved from an archive and reprocessed using the current software and a summary of all previous results included in the current report. If this is not possible, the mean difference and limits of agreement of the two software methods should be given.

The pattern of our results for reproducibility is consistent with previous reports in the literature by van Royen *et al.*,^11^ Pfistererer *et al.*,^12^ , Hains *et al.*,^13^ Hiscock *et al.*,^3^ and Skrypniuk *et al.*^4^ Van Royen *et al.*^11^ found that repeat quantitative radionuclide assessments of LVEF can be expected to be within a 2–4% range if a study is processed twice by the same operator. Pfisterer *et al.*^12^ do not state how many times each study was processed, but found studies reprocessed by the same operator to be within a 1–3% range of each other and within a 1.4–5% range of each other if processed by different operators.

We found that studies processed three times by the same operator were within a 3–6% range of each other for the Siemens method, and within a 3–9% range for the Hermes method. The reason for the wider range in our study is most probably due to the fact that our studies were processed three times. Two of our three estimates were always more closely related. The difference between the two closest estimates was ≤ 3% in all patients with both methods.

Our study is in agreement with that done by Hains *et al.*^13^ were the SD of the difference between estimates of LVEFs calculated by one operator ranged from 0.5–0.8. Hiscock *et al.*,^3^ who also compared the Hermes and Siemens systems, reported the SDs of the difference between LVEFs calculated by one operator to be 1.80 and 2.56 for Hermes software systems. Their reported value for inter-operator variability for the Siemens software system, 4.79, was however much higher than the values of 0.6, 0.7 and 0.5 reported in our study for the three operators, respectively. The reason for the higher value is not known. Skrypnuik *et al.*^4^ reported the SD of the difference between LVEFs calculated by one operator to be 0.002.

To date no articles on the influence of the acquisition of GBP studies on different cameras could be found in the English literature. In a study on the intra- and inter-observer reproducibility of LVEF estimates obtained from gated myocardial perfusion SPECT imaging and those obtained from GBP imaging, Castell-Conesa *et al.*^14^ collected data from two institutions, which acquired their data on different cameras. The issue of whether the acquisition of studies on different cameras had an effect on results was however not specifically addressed. Our study showed no difference in the results of the GBP studies acquired on different cameras.

We suggest that each GBP study should be processed three times before reporting a result. In our study, two of the three LVEF estimates were closely related regardless of the software method used. The difference between the two closest estimates was always ≤ 3%. The mean of these two estimates would probably be the best representation of the patient’s LVEF.

Anthracyclines have been used for the past 30 years in chemotherapy regimes. No consensus however exists on the optimal monitoring for cardiotoxic effects. Guidelines have been proposed, but none incorporate all of the necessary components of monitoring for chemotherapy-induced cardiotoxicity.^15^ Until such research is available, following one of the existing guidelines is the most practical solution.

At our institution the oncologists use the guidelines as set out in the *Oxford Textbook of Oncology*.^16^ This guideline suggests that in patients with baseline LVEF estimates greater than 50%, doxorubicin treatment should be discontinued if the LVEF decreases by 10%, or if a value of 50% is reached. In patients with baseline LVEFs of less than 50% but greater than 30%, it is suggested that doxorubicin therapy should be discontinued if the LVEF decreases by 10% or if a value of less than 30% is reached.

Skrypniuk *et al.*^4^ found the change in a LVEF value required to be 95% confident of a real change when carrying out repeat measurements on an individual patient to be 4.5%. It is therefore recommended that each department obtain their own percentage values for clinical decision-making for each software package used independently.

In our study, the patients who had SDs for the three estimated LVEFs above the 95th percentile had differences between the highest and lowest (maximum difference) of the three estimates of 6% using the Siemens software method, and 9% using the Hermes software method. This implies that within our department, a reduction in the ejection fraction for a follow-up study of more than 6% is of clinical significance if processed using the Siemens software method, and 9% when using the Hermes software method. If the closest two of the three estimates of LVEF obtained by one operator is used, changes of more than 3% are of concern.

A limitation of this study was that most of the LVEFs fell within the normal range.

## Conclusion

The results of this study are consistent with reports that software programs for processing GBP studies cannot be used interchangeably and each department must establish its own values for clinical decision-making. The software used to calculate the result should be identified in the report. Studies should be processed at least three times. The mean of the two closest estimates would probably be the best representation of the patient’s LVEF. Acquisition of GBP studies on different cameras did not influence results.
